# Innovative Purification Method of Ovatodiolide from *Anisomeles indica* to Induce Apoptosis in Human Gastric Cancer Cells

**DOI:** 10.3390/molecules27030587

**Published:** 2022-01-18

**Authors:** Hsiu-Man Lien, Shiau-Huei Huang, Chi-Huang Chang, Chao-Lu Huang, Chia-Chang Chen, Charng-Cherng Chyau

**Affiliations:** 1 Research Institute of Biotechnology, Hungkuang University, Shalu District, Taichung 43302, Taiwan; lien736@gmail.com (H.-M.L.); a7651618@yahoo.com.tw (S.-H.H.); ok1456@hk.edu.tw (C.-H.C.); 2 Yusheng Biotechnology Center, No. 26, Keyuan Rd., Xitun District, Taichung 40763, Taiwan; chaoluhng@gmail.com (C.-L.H.); casey5115@gmail.com (C.-C.C.)

**Keywords:** *Anisomeles indica*, ovatodiolide, centrifugal partition chromatography, human gastric cancer cell line AGS, apoptosis

## Abstract

Ovatodiolide (Ova), found in the plant *Anisomeles indica* (AI), has been reported to have an anti-proliferation effect in various cancer cells. However, little information is available regarding the anti-cancer effect of Ova in human gastric cancer cells. In this study, we investigated the inhibitory effects and the mechanisms of action responsible for these effects on human AGS cell lines from a newly developed purification technique for Ova from AI extract. Extract obtained at the optimum condition of 95% ethanol extraction of AI was sequentially partitioned by using different polarity solvents. Enriched content of Ova (35.9% purity) from the *n*-hexane fraction was then applied to the purification by using centrifugal partition chromatography (CPC) in a two-phase solvent system consisting of *n*-hexane:ethyl acetate:methanol:water (1.0:1.0:1.0:1.0, *v*/*v*/*v*/*v*) to reach purity over >95.0%. In evaluation of the anti-proliferation effect on AGS cells, Ova induced cell apoptosis with IC_50_ values of 13.02 and 6.18 μM at 24 and 48 h, respectively, and arrested the cells at the G2/M phase. Quantification of Bax/Bcl2 mRNA expressions using qPCR showed a 2.5-fold increase in the Ova (5 μM)-treated cells at 48 h than in the control group. Specific protein expression data warrant further research to further confirm the proposed Ova-induced apoptotic pathway in AGS cells.

## 1. Introduction

Ovatodiolide (Ova), a cembrane-type diterpenoid, is one of the well-known secondary metabolites produced from *Anisomeles indica* (L.) Kuntze (AI). In traditional medicine, aerials parts of the plant are used for treatments of gastrointestinal disease, inflammatory skin disorder, immune system deficiencies and hypertension [1,2]. According to the review of ethnomedicinal properties in Nepalese medicinal plants, AI has been explored for the treatment of urinary affections [3]. This evidence has indicated that the broad bioactivities of AI are worthy of further study.

The report from the International Agency for Research on Cancer (IARC) in 2018 indicates that gastric cancer is the third leading cause of cancer deaths worldwide [4]. Although surgical resection is a possible curative treatment in cases of gastric cancers, medicines are still the first favorable choice for treatment. Unfortunately, the therapies of medicines available to date for advanced and metastatic gastric cancer do not present clear survival benefits [5]. Traditional herbal medicine possesses years of experience in both disease prevention and treatment. The experiences of AI applications have drawn much attention due to their extensive biological activities. In recent decades, Ova has been the most valued compound for anti-inflammation and anti-cancer studies on AI. Ova possesses anti-inflammatory activity [6], anti-HIV activity [7] and therapeutic potential for the treatment of allergic asthma [8]. Recently, emerging evidence of Ova-induced apoptosis in the treatment of human lung cancer A549 and H1299 cells, breast cancer AS-B145 and BT-474 cells, oral squamous cell carcinoma SCC-15 and CAL27 cells, and renal cell carcinoma 786-O and A498 cells has been revealed through different pathways [9,10,11,12]. An Ova-mediated cytotoxic effect on A549 lung cancer cells has been indicated by a decrease in Bcl-2 and increase in PUMA, DR5 and Bax protein levels, as well as activation of caspase cascades [9]. In breast cancer stem/progenitor cells (CSCs), Ova presents inhibitive activity through SMURF2-mediated downregulation of Hsp27 [12]. Based on the aforementioned evidence, we suggest that Ova might be a potential candidate in the treatment of gastric cancer cells.

**Figure 1:**
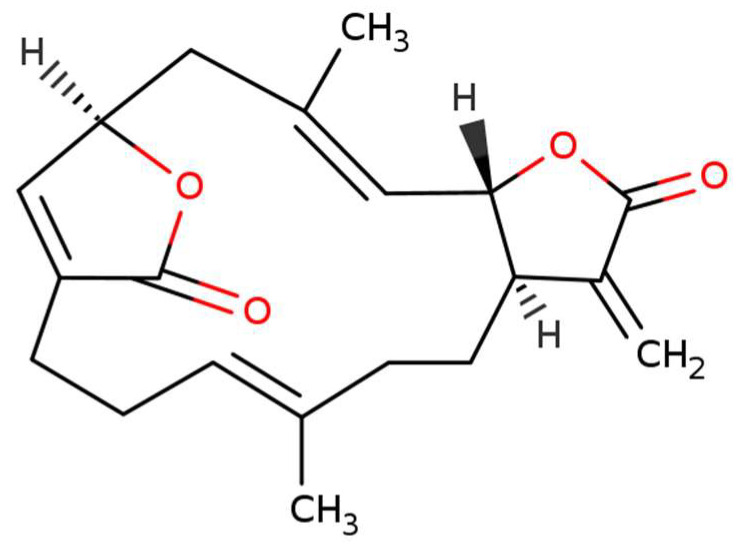
Chemical structure of ovatodiolide (the U.S. National Library of Medicine, RN: 3484-37-5).

Cembrane diterpenoids have been reported with diverse structural variations with different functional groups (lactone, epoxide, furan, ester, aldehyde, hydroxyl, carboxyl groups) and cyclizations [13]. Ova is characterized as a cembrane diterpenoid with a five-membered lactone and is referred to as a hydrophobic molecule (Figure 1). Thus, the extraction of cembranoid diterpenes is generally conducted with organic solvents, such as ethanol, ethyl acetate, *n*-butanol, etc. [13]. Pure Ova is still unavailable in the pharmaceutical ingredients market. Owing to the significant contribution of Ova to anti-cancer studies, a high-purity Ova standard has now become a major issue medical studies. As stated in a previous report on the preparation of ovatodiolide, solvent extraction was applied and followed by tedious column separation methods, such as Celite 545 column and silica gel column chromatography [14]. Although column chromatography has been widely applied to the purification of cembrane diterpenoids [13], the disadvantages of solvent extraction and tedious purification procedure have prompted us to develop a concise and effective method for purification work. Recently, centrifugal partition chromatography (CPC) has become an alternative method developed to replace the resin adsorption methods. CPC is derived from countercurrent chromatography (CCC) based on the similar liquid–liquid partition principles [15]. In CPC processing, there is no solid support required for the separation of constituents in samples and non-irreversible adsorption of targets, with many advantages over traditional liquid chromatography [16].

The aim of this study was to establish an efficient preparation of the anti-cancer active compound Ova from AI extract using CPC. In view of our previous findings of inhibitive activities of Ova on *Helicobacter pylori*-induced inflammation in gastric epithelial cells [17], whether Ova can inhibit the proliferation of AGS cells (a human gastric adenocarcinoma cell line) would be expected. For a preliminary study of anti-gastric cancer activity of Ova, we focused on the investigation of expression behaviors of the apoptosis-related genes Bcl-2, Bax and caspase-3 induced by the treatment with Ova. We propose that AGS cells could be effectively inhibited by Ova via modulating the pro-apoptotic-related genes, and anticipate that the preliminary mechanism of action might support intensive study in the future.

## 2. Results

### 2.1. Extraction Yields and Ovatodiolide Contents of the AI Extract

**Figure 2:**
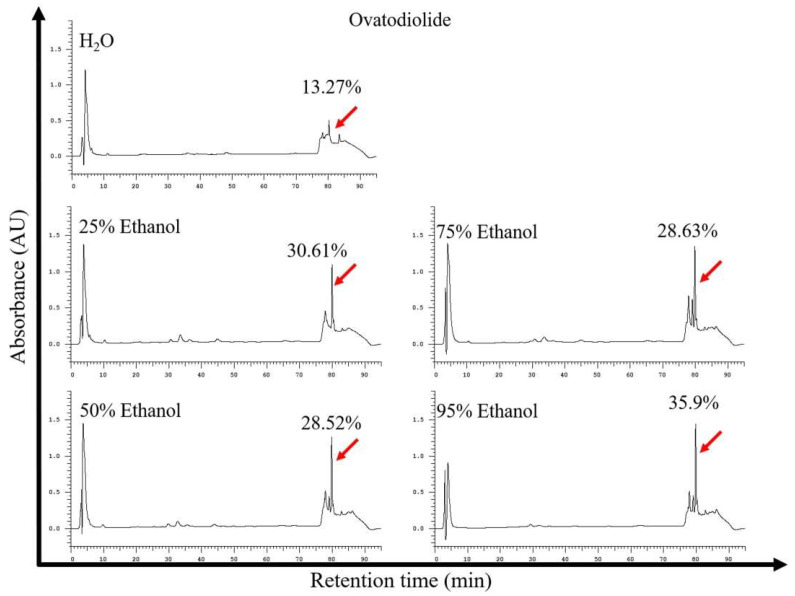
HPLC profiles of extracts from the extractions of water and hydroethanol solution from *Anisomeles indica*. Analysis column: Luna C18, 150 × 2 mm, 3 µm. The highest amount (35.9%) of Ova in crude extract was found in solvent extraction using 95% ethanol AI.

The yield of aqueous and hydroalcoholic extraction with different proportions of ethanol:water mixtures from AI is shown in Table 1. The extraction using the solvent of 50–75% ethanol obtained the highest yield with a total of 21.26–22.57% (*w*/*w*). However, the Ova contents in 50–75% ethanol extracts were found only in 28.52–28.63% lower than that extract obtained from 95% ethanol extraction (Figure 2). The 95% ethanol extraction further improved the content of Ova in the extract to 35.9% (Figure 2). Obviously, the extraction of 95% ethanol solvent appeared to be the best extraction solvent to recover Ova.

### 2.2. Ovatodiolide Content in Different Solvent Partitions of 95% Ethanol Extract

From an ideal purification point of view, the factors that affect the choice were the yield and the content of the target compound in the extracts. In the study, we determined the 95% ethanol extract as the candidate for purification of the target compound Ova owing to the acceptable yield and the fewer impurities for omitting complicated purification procedures. For enriching the content of Ova in extracts, the 95% ethanol extract of AI was further fractionated by using solvent partitions in different polarities of solvents. As a result, the highest content of Ova in the four partitions was obtained from the ethyl acetate partition (11.76%) followed by the *n*-hexane partition (11.26%). However, the partition yield from the *n*-hexane partition (2.87%) was significantly higher than that of the ethyl acetate partition (1.42%) (Table 2). It is worth mentioning that even the highest yield of the partitions was obtained from the *n*-butanol partition (4.70%), however, the low content (6.94%) of Ova (Table 2) and highly complicated constituents in the partition (data not shown) gave the partition a disadvantage when following the purification procedure. Thereafter, the *n*-hexane partition was selected as the candidate for the following CPC purification study.

### 2.3. Ovatodiolide Purification by Ascending Mode of CPC

For successful separation of target compounds in CPC, the partitioning condition of target compounds in the solvent system is critical for appropriately isolating targets from the mixtures. At the beginning, an optimal two-phase solvent system with an appropriate partition coefficient (K) was developed for CPC processing. The suitable two-phase solvent system consisting of *n*-hexane-ethyl acetate:methanol:water was determined, as shown in Table 3. The solvent ratio of *n*-hexane-ethyl acetate:methanol:water (1.0:1.0:1.0:1.0, *v*/*v*/*v*/*v*) seemed to be suitable for separation of Ova. As a suitable K value should be between 0.5 and 3.0 [15], an ascending mode of a Ka value of 0.7 (Table 3) in the solvent system was determined to be applied in the CPC study.

**Figure 3:**
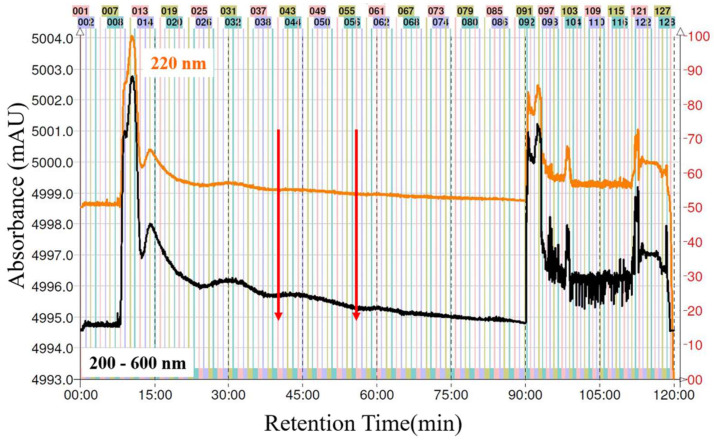
The CPC–UV chromatogram of ovatodiolide prepared from the *n*-hexane fraction of 95% ethanol extract of AI. Two-phase solvent system: *n*-hexane:ethyl acetate:methanol:water (1.0:1.0:1.0:1.0 *v*/*v*/*v*/*v*); mobile phase: the lower aqueous phase at flow rate of 10 mL/min for 2.5 h; stationary phase: upper organic phase, same flow rate for 2.5 h. Eluted compounds were monitored at 222 nm. Ova fraction was collected as indicated by the vertical red lines No. 41–56.

In running CPC with an ascending mode, the column was filled with the lower stationary phase, and the upper phase was used as the mobile phase. The column was first entirely filled with the lower phase. Subsequently, the upper mobile phase was then pumped into the inlet of the column as the mobile phase at a flow rate of 10 mL/min, and the sample from the *n*-hexane partition (10 mg/mL) was then injected into the system. As shown in Figure 3, the Ova collected in tubes No. 41–56 were combined, concentrated in vacuo and freeze-dried. Finally, Ova was obtained at a purity of 95.6%, with a yield of 0.29 ± 0.02 mg/g from dried powder of AI.

### 2.4. HPLC and LC/MS Determine the CPC Purified Ovatodiolide

**Figure 4:**
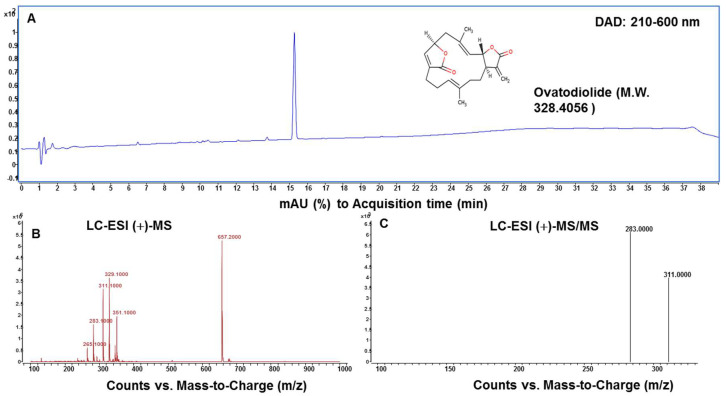
HPLC profile and mass spectra of ovatodiolide isolated from AI by using CPC. Conditions: UV-Vis spectrum detection at 210–600 nm (**A**); HPLC-ESI(+)-MS analysis (**B**) and product ions analysis from collision-induced dissociation of molecular ions at *m*/*z* 329 (**C**). Analysis column: HSS-T3 C18 3 µm, 100 × 2.1 mm i.d.

The structural identification of isolated compound Ova was performed with HPLC and LC/MS-MS analysis and comparison with published MS data. As shown in Figure 4, the purity of Ova was more than 95% after the HPLC analysis (Figure 4A). The identification data of the molecular ions (Figure 4B) and MS/MS spectra of Ova agreed with the authentic compound which has been published in our previous study [17].

### 2.5. Inhibition Rate of 95% Ethanol Extract on Gastric Cancer AGS Cells

**Figure 5:**
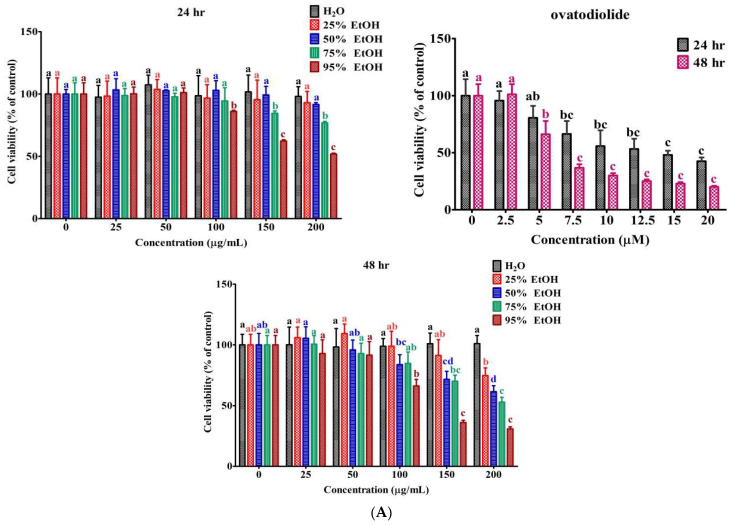

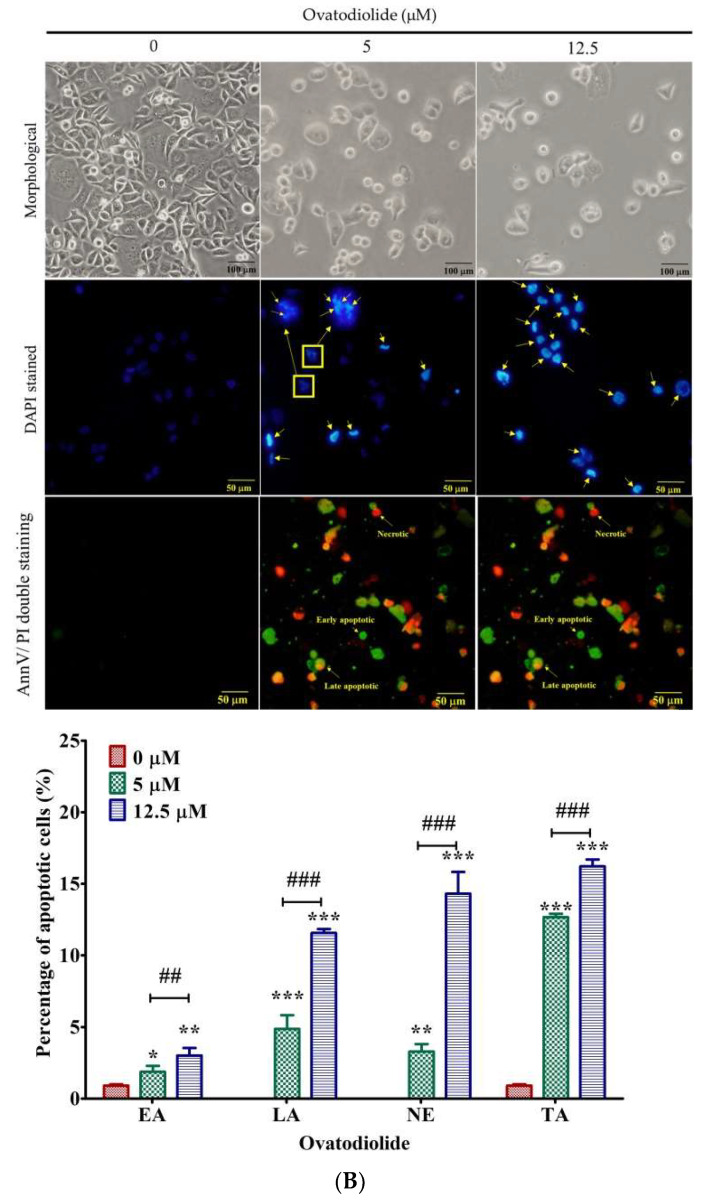
Effects of ethanolic extract and ovatodiolide on cell viability and apoptosis in AGS cells. (**A**) Inhibition of the growth by various ethanolic extracts and purified Ova from *Anisomeles indica* in AGS cells. AGS cells were exposed to ethanolic extract and Ova at the indicated concentrations for 24 and 48 h, respectively, followed by MTT assay. Results are presented as the mean ± SD (*n* = 8). Letters indicate significant differences (*p* < 0.05) between concentrations tested for each phase of the cell cycle by Tukey’s multiple comparison test. (**B**) Morphology of AGS cells after treatment with Ova. Cells were grown and treated with Ova for 24 h. The medium was removed and attached cells were fixed with 4% paraformaldehyde and washed with PBS. Cell morphology was examined by light microscope (viewed at magnification of 100×, upper panel) and stained with DAPI (middle panel) and Ann V/PI (bottom panel) in PBS for 10 min in the dark and nuclear condition was detected by fluorescence microscope (viewed at magnification of 200×). Arrows indicate cell shrinkage and nuclear fragmentation (DAPI staining). Ann V/PI staining counts presented the differences among the early (Ann V ^pos^/PI ^neg^, EA), late (Ann V ^pos^/PI ^pos^, LA) apoptotic, necrotic (Ann V ^neg^/PI ^neg^, NE) and total apoptotic (TA) cells in the treatments with Ova at 0–12.5 μM (the bottom right panel). * *p* < 0.05; ** *p* < 0.01; *** *p* < 0.001 compared to 0 μM; ^##^ *p* < 0.01; ^###^ *p* < 0.001 compared to 5 μM.

In order to evaluate the effect of various ethanolic extracts of AI on the proliferation of AGS cells, the cells were treated with various concentrations of the extract for 24 and 48 h, respectively, followed by an MTT assay. The results (Figure 5A) indicated that the 95% ethanolic extract significantly (*p* < 0.05) showed the best results by effectively inhibiting the cell growth of cells compared to 0–75% ethanol extracts in a dose-dependant manner at concentrations of 0–200 μg/mL for 24 and 48 h, respectively, whereas the water extract did not reveal any obvious toxicity on cell growth in either 24 or 48 h treatments. Considering more considerable cytotoxic effects of 95% ethanol extract on AGS cells, active compounds with hydrophobic chracterisctics would be extracted in a high concentration of ethanol.

The results showed that Ova inhibited the proliferation of AGS cells in a dose-responsive manner with the IC_50_ value of 14.06 μM at 24 h. Moreover, the IC_50_ values of Ova and 95% ethanol extract were 6.79 μM and 126 μg/mL at 48 h, respectively, as shown in Table 4. Hence, it can be supposed that 95% ethanol extract showed preferential cytotoxicity against AGS cells. In subsequent experiments, the extract was applied to the partition and purification of the active compound.

### 2.6. Ovatodiolide Induces Mmorphological Changes and Apoptosis in AGS Cells

DAPI staining was used for assessing the changes in nuclear morphology. The fluorescent blue nuclei shown in Figure 5B (middle panel) presented the characteristic of nuclear condensation and possible apoptosis induced by ovtaodiolide in a dose-dependent manner. Furthermore, AnnV/FITC and PI staining was applied to detect apoptosis and necrosis. As shown in Figure 5B (lower panel), the AGS cells treated with Ova at concentrations of 0–12.5 μM for 24 h treatments showed a significantly increased proportion of apoptotic cells in comparison with the control cells, indicating that ovatodiolide induced apoptosis.

### 2.7. Ovatidiolide Induces Cell Cycle Arrested at G2/M Phase

**Figure 6:**
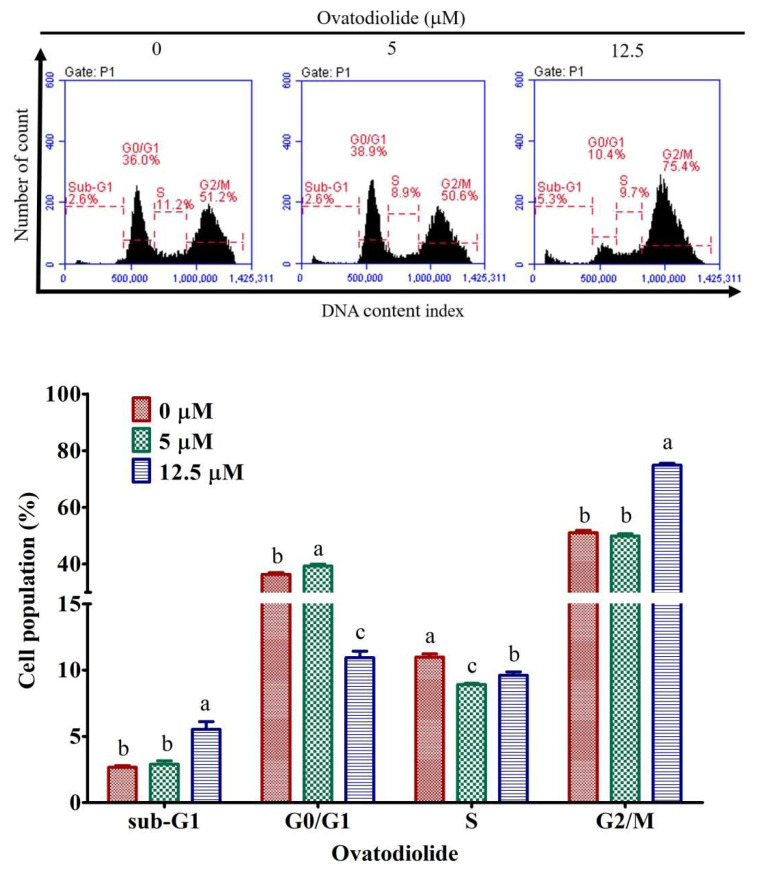
Effects of Ova on cell cycle progression and apoptosis in human gastric cancer cell lines. Cells were exposed to Ova at the indicated concentrations or to vehicle control (0 μM) for 24 h. Results are presented as the mean ± SD (*n* = 3). Letters indicate significant differences (*p* < 0.05) between concentrations tested for each phase of the cell cycle by Tukey’s multiple comparison test.

Since Ova inhibited AGS cell growth, the cell cycle arrest in Ova-treated cells was analyzed by a flow cytometer. After treating cells with dimethyl sulfoxide (DMSO) or 5 and 12.5 μM Ova for 24 h, flow cytometry analysis was performed. Ova (7.5–12.5 μM) caused a marked increase (up to ~24%) in the G2/M fraction of cells and also induced accumulation of some cells in the G0/G1 phase at a lower concentration (5 μM) (Figure 5A) compared to the control (Figure 6, bottom panel), and simultaneously decreased the cell population in the G0/G1 phase (up to ~25%). As described above, Ova increased the frequency of cells containing chromatin condensation and apoptotic body formation from the results of DAPI staining (Figure 5B).

### 2.8. Expression Levels of Bcl-2, Bax and Caspase-3 Genes Are Modulated by Ovatodiolide

**Figure 7:**
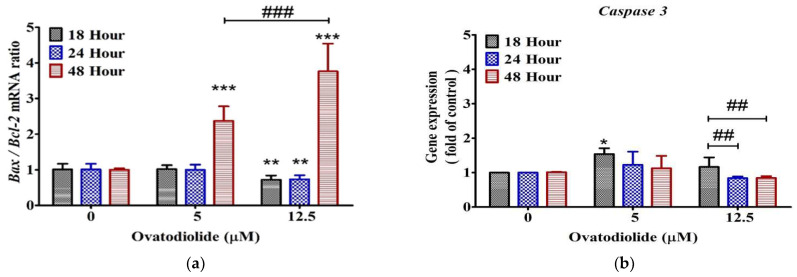
Effect of ovatodiolide on relative mRNA levels of *Bax*, *Bcl-2* and *caspase-3* genes in controls versus ovatodiolide-treated groups at 18, 24 and 48 h, respectively. The quantified mRNA of Bax/Bcl2 (**a**) and caspase-3 (**b**) in AGS cell line was measured by RT-PCR. ANOVA was followed by Dunnett’s multiple-comparison post-test. * *p* < 0.05, ** *p* < 0.01, *** *p* < 0.001 vs. control; ^###^ *p* < 0.001, ^##^ *p* < 0.01. Each bar represents the mean ± SD (*n* = 3).

Nuclear condensation induced by Ova in AGS cells prompted further investigation of apoptosis. The expression levels of the apoptosis-related genes *Bcl-2*, *Bax* and *caspase-3* were studied and quantified in AGS cells through qPCR by using a highly sensitive fluorescence detection of SYBR Green I. The results showed that the relative mRNA expression levels of *Bax* to *Bcl-2* (*Bax*/*Bcl-2*) presented a significantly increasing trend in the Ova group compared with the control in time- and dose-responsive manners (Figure 7, left panel). Unexpectedly, the mRNA levels of *caspase-3* remained unchanged at 24–48 h in the Ova-treated groups as compared to the control group (Figure 7, right panel). However, there were still partial impacts of mRNA levels of *caspase-3* in the treatment of 5 μM at 18 h.

## 3. Discussion

*Anisomeles indica* (AI), a commonly used medicinal herb, has been reported to have anti-inflammatory activity in treating gastrointestinal diseases [2]. In addition, the anti-cancer activities of AI and its active compounds on various cancer cells have also been published [1,9,10,11,12]. *Anisomeles* is a genus of herb of the family Lamiaceae. The previous reports have indicated that the same cytotoxic properties in the induction of apoptosis in AGS cells of nanoparticles prepared from the *Artemisia turcomanica* and *Artemisia ciniformis* extracts [18,19]. However, there have been no studies to date developing a simple and efficient purification method for the active compound of Ova of the plants and on the cytotoxic activities on gastric cancer cells at the same time. Ova showed very potent activity on AGS cells with IC_50_ values of 13.02 and 6.18 μM at 24 and 48 h, respectively (Table 2), as compared to the cytotoxicity on human breast cancer cell lines AS-B145 and BT-474 with IC_50_ values of 6.55 and 4.80 μM, respectively, at 72 h [12]. However, the significant anti-cancer effects attract attention to the safety of Ova applied in the treatment of relevant diseases. In the latest research report, it has been demonstrated that Ova does not display any mutagenicity or genotoxicity. Moreover, repeated oral dosing in rats once a day for 14 days had no obvious morbidity or clinical signs of toxicity after administration of Ova in a single dose of 1000 mg/kg b.w. [20]. The proved safety of Ova in developing medical and pharmaceutical applications was highly expected.

It has been suggested that gene expression is tightly linked to our understanding of protein expression. These gene and protein expression studies offer determination and prognostication in cancer treatments [21]. To further investigate the underlying mechanisms of anti-cancer activity of Ova, we briefly compared three important indicators from mRNA expressions in evaluating the possible inhibition activities on AGS, including *caspase-3*, *Bcl-2* and *Bax*. Caspase-3 has been recognized as one of the apoptosis executioners, while BCL-2 is known for anti-apoptosis and Bax for promoting cell death. One of the important findings in cancer studies is that the ratio of pro-apoptotic (Bax) to anti-apoptotic (Bcl-2) proteins can be a key checkpoint in the common portion of the cell death signal pathway [22,23,24]. In a similar study from Yu et al. [9], they revealed that Ova induces the apoptosis of lung cancer cell lines A549 and H1299 through both intrinsic and extrinsic pathways characterized by elevated PUMA, Bax and DR5 proteins, decreased Bcl-2 and Mcl-1 and activated caspase-8, caspase-9 and caspase-3. In a clinical study, Gryko et al. [25] found positive expression of Bcl-2 protein in 55.7% of patients with gastric cancers. Moreover, a significantly higher frequency of anti-apoptotic Bcl-2 expression in patients with more advanced tumors as compared to an earlier stage was also observed in the study [25]. When the apoptosis-inducing factors (the Bcl-2 family, whose members include Bax, Bik, Bad, Bid, Bim, Bcl-2, Bcl-x, etc.) are activated, the apoptosis mechanism will be started. Under the influence of apoptosis-inducing factors, mitochondria will release cytochrome C to interact with caspases and bind to apoptosis protein activating factor 1, and then activate downstream apoptotic proteases (such as caspase 9 and caspase 3) to promote apoptosis [26]. Thus, the expression capacity of Bcl-2 of cancer cells may be recognized as one of the key indicators for cell growth after treatment with Ova. Similar studies on Ova from AI have been reported, including anti-cancer properties against human oral squamous cell carcinoma cells in a concentration–response relationship. The IC_50_ values of Ova are 4.4 and 2.5 μM at 24 and 48 h after treatment, respectively [27]. Meanwhile, Ova presents better inhibition effects against the cancer cell growth than cisplatin and 5-FU with IC_50_ values of 77.0 and 46.9 μM at 48 h, respectively [27]. A recent study indicates that Ova inhibits colon cancer tumor growth via downregulating IL-6, STAT3 and β-catenin expression and serum exosomal miR-1246 in mouse xenograft studies [28]. These results indicate that Ova can exert inhibition effects on cancer cell growth by altering the cell cycle and upregulating apoptosis-inducing potential in the cancer cells. Hence, the preliminary results suggest that Ova can induce cell apoptosis through the intrinsic pathway by increasing the ratio of *Bax* to *Bcl-2* and the level of *caspase-3* to regulate the cell cycle arrest.

The question now arises whether the obstacle of pilot-scale preparation of Ova may be conquered. CPC is an emerging separation technology in the purification of natural products. The advantages of CPC are the support-free liquid stationary phase used in the separation of mixtures without the disadvantages of the sample’s irreversible adsorption and contamination in comparison with the conventional column chromatography [29,30,31]. Although the liquid–liquid extraction and partition chromatography processes in CPC have been indicated to significantly improve the separation efficiency and reduce the preparation time [29,32,33], automation and continuity are highly required in the preparation and recycling of the solvent wastes for economical CPC operation [33]. In addition, the process of defining the conditions of CPC when choosing solvent systems is often time-consuming [15,33,34]. In the study, we have established for the first time the CPC conditions of the purification of Ova. The next step would be developing the continuous process for purification and scaling up the preparation of Ova for a preclinical study.

## 4. Materials and Methods

### 4.1. Chemicals

The MTT [3-(4,5-dimethylthiazol-2-yl)-2,5-diphenyltetrazolium bromide], 4′,6-diamidino-2-phenylindole (DAPI) and Ham’s F-12K medium were purchased from Sigma-Aldrich (St. Louis, MO, USA). Acetonitrile, methanol, n-butanol and ethyl acetate were of HPLC grade and supplied either by Sigma-Aldrich (St. Louis, MO, USA) or Merck (Kenilworth, NJ, USA). An annexin V fluorescein isothiocyanate (FITC) apoptosis detection kit was purchased from BD Pharmingen (San Diego, CA, USA). Fetal bovine serum (FBS) and antibiotics (penicillin/streptomycin) were purchased from Gibco (BRL Life Technologies, Grand Island, NY, USA).

### 4.2. Plant Material and Extraction

The fresh plants of *Anisomeles indica* (AI) were obtained from contractual farms located at Taichung, Taiwan during the harvest season of winter (November to December, 2020). After being air- and oven-dried (50 °C) and pulverized (40 mesh), the dried powder was then stored at −30 °C for further extraction.

**Figure 8:**
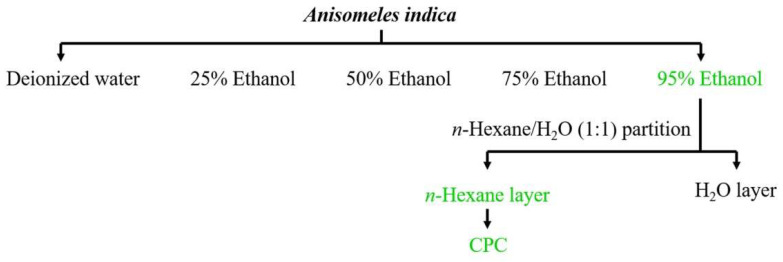
The flow chart of solvent extraction and partitioning for isolation of ovatodiolide from *Anisomeles indica*.

The extractions of oven-dried powder and partitioning of extracts were conducted with the procedures shown in Figure 8. In brief, ten grams of powder was extracted with 100 mL of distilled water (dw) or ethanol/dw mixture in different proportions in triplicate extraction under sonication (Delta Sonicator DC200H, LMI Co. Ltd., Taipei, Taiwan) at room temperature for 30 min. The obtained extracts were concentrated under vacuum by a rotary vacuum evaporator ((Rotavapor R-114, Büchi Labortechnik AG, Flawil, Switzerland) at 40 °C. The highest content of Ova from 95% ethanol extract was further submitted to liquid–liquid partitioning sequentially using *n*-hexane, ethyl acetate and *n*-butanol as in a previous report [35] to obtain *n*-hexane, ethyl acetate, n-butanol and aqueous fractions, respectively. The obtained *n*-hexane fraction containing the highest amount of Ova was further purified by using CPC.

### 4.3. HPLC-MS Determination of Ovatodiolide in Crude Extracts and Partitions

The determination of Ova was carried out by an Agilent 1200 HPLC system connecting a Security-Guard Ultra C18 guard column (2.1 mm × 2.0 mm, sub-2 µm, Phenomenex, Inc., Torrance, CA, USA) and an analytical C18 column (Acquity UPLC HSS T3. 2.1 × 100 mm, 1.8 μm, Waters Corporation, Milford, MA, USA) in a column oven set at 35 °C. The mobile phase consisted of two solvents: Solvent A (water containing 0.1% formic acid) and Solvent B (acetonitrile containing 0.1% formic acid). The flow rate during the elution process was set at 0.3 mL/min. A gradient elution was carried out with 10% B for 0–3 min, 10–40% B for 3–20 min, 40–95% B for 20–25 min and finally 95% B isocratic elution for 10 min. The column was kept in a column oven at a constant 35 °C for all experiments. The absorption spectrum of the eluted compound was scanned within 210 to 600 nm using the in-line PDA detector monitored at 222, 254, 280 and 325 nm, respectively. The HPLC-triple quadrupole mass spectrometry system (Agilent 6420, Santa Clara, CA, USA) was used to confirm the compositions of extracts for which compounds were eluted and separated. The nitrogen gas acted with both functions, as the drying gas controlled at a flow rate of 9 L/min, and as a nebulizing gas operated at 35 psi. The drying gas temperature was 325 °C, and a potential of 3500 V was applied across the capillary. The fragmentor voltage was set at 130 V, and the collision voltage, 15V. The quadrupole 1 filtered the calculated m/z of each compound of interest, while quadrupole 2 scanned for ions produced by nitrogen collision of these ionized compounds in the range 100–1000 m/z at a scan time of 200 ms per cycle. Mass data were acquired in positive ionization mode. The identification of separated compounds was carried out by comparing their mass spectra provided by ESI-MS and ESI-MS/MS from the identified authentic compound as previously described [17].

### 4.4. CPC Procedure in the Purification of Ovatodiolide

CPC experiments were performed on an Armen fully integrated Spot Prep instrument (Armen Instruments, Saint-Avé, France). The instrument is a fully automated system equipped with a 250 mL rotor containing 8 stacked disks in a total of 576 twin-cells, which were connected with passages to each other; each chamber has a volume of 0.217 mL with column capacity of 250 mL. The CPC columns were coupled with a Spot Prep I (Armen Instruments) integrated preparative HPLC instrument equipped with a built-in two-headed quaternary gradient HPLC pump, an injector loop (10 mL), a Flash 10 DAD 600 detector (Ecom, Prague, Czech Republic), an automatic fraction collector, a digital screen and a flat panel PC with Armen Glider software. A solvent system consisting of *n*-hexane:ethyl acetate:methanol:water in different ratios (Table 3) was applied in the CPC separation. The mobile phase: the lower aqueous phase at a flow rate of 10 mL/min for 2.5 h; stationary phase: upper organic phase, same flow rate for 2.5 h. Eluted compounds were monitored at 222 nm.

### 4.5. Effects of Ovatodiolide on AGS Gastric Cancer Cell Growth

The AGS cells (purchased from the Biosource Collection and Research Center (BCRC), Hsinchu, Taiwan), a human gastric adenocarcinoma, were grown in Ham’s F-12K medium (Sigma-Aldrich, St. Louis, MO, USA) containing 10% fetal bovine serum (Gibco, USA) in 5% CO_2_ at 37 °C. The cytotoxicity effects of AI extracts and Ova on AGS cells were measured using the MTT [3-(4,5-dimethylthiazol-2-yl)-2, 5-diphenyltetrazolium bromide] assay. The cells were seeded at a density of 5 × 10^3^ cells per well into 96-well culture plates overnight and treated with various concentrations of the extracts (25~200 μg/mL) and Ova (2.5~20 μM). After 24 and 48 h of incubation, 10 μL of MTT (5 mg/mL) was added to each well followed by 2 h of incubation at 37 °C. Consecutively, the supernatants were removed and 100 μL DMSO was added to wells and plates were shaken for 10 min. The absorbance was measured at 570 nm by a microplate reader (ELx800, BioTek Instruments Inc., Winooski, VT, USA). The cell viability was assessed at 24 h and 48 h.

### 4.6. DAPI and Annexin V/Propidium Iodide (AnnV/PI) Staining

For the nuclear morphology (DAPI staining) assay, AGS cells (2 × 10^5^ cells per well) were treated with or without ovatodiolide at 37 °C for 24 h, washed with PBS and fixed with 4% paraformaldehyde, re-washed with PBS and incubated with DAPI (1 μg/mL in PBS) for 10 min. Cells were examined to assess changes in nuclear morphology and photographed under a fluorescence microscope (Olympus, Tokyo, Japan).

AnnV/PI staining was also used to identify apoptotic cells. In brief, the AGS cells were subjected to treatment with Ova (0, 5, 12.5 μM) and were incubated for 24 h. The cells were suspended in trypsin–EDTA and centrifuged to obtain cell pellets. The pellets were then washed in cold PBS (0.5 mL) and pelleted by centrifugation (1000 rpm, 5 min). After removal of the PBS, the cells were resuspended in the staining solution (1 × binding buffer 400 μL, propidium iodide 5 μL, Ann V-FITC 5 μL) and incubated in the dark for 30 min, followed by subjection to fluorescence microscope examinations. After staining a cell population with Ann V and PI, morphological patterns of apoptotic cells show green fluorescence, dead cells show red and green fluorescence and live cells show little or no fluorescence [36]. A minimum of 300 cells were counted in each sample [37].

### 4.7. Flow Cytometric Analysis of Cell Cycle

AGS cells (4 × 10^5^) were treated with different concentrations of Ova for 24 h. After that, the cells were washed with ice-cold PBS, trypsinized, collected in a 15 mL conical tube and pelleted by centrifugation 1200 rpm for 3 min. The pellets were washed twice with ice-cold PBS and fixed in 70% cold ethanol at −20 °C for 3 h. The cells were centrifuged again to remove the supernatant and 500 μL of PI staining solution consisting of 0.1% Triton X-100, 50 µg/mL propidium iodide (PI) and 200 µg/mL RNase A was added into each tube. The stained cells were re-suspended in the dark at 37 °C for 30 min, followed by subjection to measurements using a BD FACS Calibur flow cytometer (Becton Dickinson, Franklin Lakes, NJ, USA).

### 4.8. RNA Isolation and Quantitative Real-Time PCR (qPCR)

To test the pro-apoptotic action of Ova, AGS cells were treated with Ova (5 and 12.5 μM) for 18, 24 and 48 h, respectively, and then real-time PCR was used to examine the gene expression levels of the BCL2-associated X protein (*Bax*), *Bcl-2* and *caspase-3*, which are important determinants of cell susceptibility to apoptosis. In brief, total RNA was extracted from 6 × 10^5^ cells using TRIzol^®^ reagent (ThermoFisher Scientific, Waltham, MA, USA), and 1.5 μg of total mRNA was reverse-transcribed using a Takara PrimeScript RT Reagent Kit (Takara Bio, Mountain View, CA, USA), following the instructions. Amplification and detection were performed with the StepOnePlus™ Real-Time PCR System (Applied Biosystems, Foster City, CA, USA). The 20 µL reaction mixture contained 9.2 µL of cDNA, 0.4 µL of 10 µM forward and reverse primers, 10 µL of KAPA SYBR FAST qPCR Master Mix (2×) (Sigma-Aldrich, St. Louis, MO, USA). The DNA fragments were amplified for 40 cycles (enzyme activation: 20 sec at 95 °C, hold; denaturation: 3 s at 95 °C; annealing: 40 s at 60 °C). The gene expression of β-actin was determined as the internal control. The relative expression level was calculated using the 2^−∆∆Ct^ method [38]. Sequences of human-specific primers for *Bcl-2*, *Bax*, *caspase-3*, and *β-actin* primers are shown in Table 5.

### 4.9. Statistical Analysis

The data were presented as a mean ± SD result and further analyzed in the GraphPad Prism program (GraphPad, San Diego, CA, USA). One-way analysis of variance (ANOVA) was used for analysis of variations in each group. Tukey’s post hoc test was used for analysis of the significance of differences among the means. A confidence level at *p* < 0.05 was considered to be statistically significant.

## 5. Conclusions

Previous studies on Ova purification from AI extract have been reported, mostly using the column chromatography method. This study highlights the efficient and rapid purification method of Ova from AI extract using CPC processing. Our findings evidenced that CPC in the ascending mode could successfully achieve increased purity from 35.9 to 95.6% after single-step purification. Purified Ova was identified by LC-MS/MS in the positive ionization mode when presented with the fragments of *m*/*z* 283, 311 from the protonated molecular ion ([M + H]^+^) at *m*/*z* 329 under collision-induced dissociation. The purified Ova significantly inhibited cell proliferation and induced cell apoptosis in a dose-dependent manner in AGS cells in association with the induction of G2/M cell cycle arrest and nuclear condensation and fragmentation. The apoptotic effects of Ova on AGS cells could be due, at least in part, to the activation of *Bax* and the reduction in *Bcl-2* mRNA levels. Further studies are required to fully investigate the underlying mechanism of cytotoxicity responsible for the anti-proliferative effects of Ova on gastric cancer cells.

## Figures and Tables

**Table 1:** The yield of different ethanolic extracts of *Anisomeles indica*.

Ethanol Concentration (%)	Yield (%, *w*/*w*) *
0	11.40 ± 1.24 ^c^
25	19.38 ± 0.13 ^b^
50	22.57 ± 0.13 ^a^
75	21.26 ± 0.84 ^a^
95	11.57 ± 0.38 ^c^

* Each value represents the mean ± SD of triplicate experiments. Statistical analysis was performed using ANOVA test, followed by Tukey’s multiple comparison test. Values with the different letters in the column are significantly different (*p* < 0.05).

**Table 2:** The content of ovatodiolide in different solvent partitions on 95% ethanol extract of *Anisomeles indica*.

Partition Solvent	Yield (%, *w*/*w*) *	Ovatodiolide (HPLC, %) *
*n*-Hexane	2.87 ± 1.06 ^ab^	11.26 ± 1.01 ^a^
Ethyl acetate	1.42 ± 0.24 ^bc^	11.76 ± 1.47 ^a^
*n*-Butanol	4.70 ± 0.88 ^a^	6.94 ± 0.56 ^b^
Aqueous	0.99 ± 0.05 ^c^	1.71 ± 0.25 ^c^

* Values with the different letters in the column are significantly different (*p* < 0.05) by Tukey’s multiple comparison test.

**Table 3:** Partition coefficient (K) of ovatodiolide measured in several different solvent systems.

Two-Phase Solvent System	Ratio (*v*/*v*/*v*/*v*)	Kd *	Ka *
*n*-hexane:ethyl acetate:methanol:water	1.0:4.0:1.0:4.0	0.30	3.36
*n*-hexane:ethyl acetate:methanol:water	1.4:3.6:1.4:3.6	0.31	3.26
*n*-hexane:ethyl acetate:methanol:water	2.0:3.0:2.0:3.0	0.16	6.09
*n*-hexane:ethyl acetate:methanol:water	1.0:1.0:1.0:1.0	1.42	0.70
*n*-hexane:ethyl acetate:methanol:water	3.0:2.0:3.0:2.0	7.17	0.14
*n*-hexane:ethyl acetate:methanol:water	3.6:1.4:3.6:1.4	0.12	8.44
*n*-hexane:ethyl acetate:methanol:water	4.0:1.0:4.0:1.0	0.12	8.55
*n*-hexane:ethyl acetate:methanol:water	4.3:0.7:4.3:0.7	0.10	10.35

* Partition coefficient of Ova was obtained from elution–extrusion techniques in descending (Kd) and ascending (Ka) modes, respectively.

**Table 4:** Growth inhibitory activities of the 95% ethanol extract and ovatodiolide on AGS cells.

Samples	IC_50_ *	
	24 h	48 h
95% ethanol extract	>200 μg/mL	126 μg/mL
Ovatodiolide	13.02 ± 3.06 μM	6.18 ± 0.80 μM

* Each value indicates mean ± SD (*n* = 3).

**Table 5:** List of primers for real-time PCR analyses.

Gene (Accession No.)	Primer (5′ to 3′)	Product Length (bp)
*Bcl-2* (NM_000633.3)	F: ACTGGCTCTGTCTGAGTAAG R: CCTGATGCTCTGGGTAAC	103
*Bax* (NM_138761.4)	F: CCCGAGAGGTCTTTTCCGAG R: CCAGCCCATGATGGTTCTGAT	155
*Caspase-3* (NM_004346.4)	F: GAAATTGTGGAATTGATGCGTGA R: CTACAACGATCCCCTCTGAAAAA	164
*β-actin* (NM_001101.5)	F: TGGCACCCAGCACAATGAA R: CTAAGTCATAGTCCGGGTAGAAGCA	186

F: forward, R: reverse.

## Data Availability

All the data on which the conclusions of the manuscript rely are presented in the paper.

## Sample Availability

Samples of the compounds are not available from the authors.
